# The Scope of Pathogenic ABCA4 Mutations Targetable by CRISPR DNA Base Editing Systems—A Systematic Review

**DOI:** 10.3389/fgene.2021.814131

**Published:** 2022-01-27

**Authors:** Elena Piotter, Michelle E. McClements, Robert E. MacLaren

**Affiliations:** ^1^ Nuffield Laboratory of Ophthalmology, Department of Clinical Neurosciences, University of Oxford, Oxford, United Kingdom; ^2^ Oxford Eye Hospital, Oxford University Hospitals NHS Trust and NIHR Biomedical Research Centre, Oxford, United Kingdom

**Keywords:** ABCA4, stargardt, adenine base editing, cytosine base editing, gene tharapy

## Abstract

Stargardt macular dystrophy (STGD1) is the most common form of inherited childhood blindness worldwide and for which no current treatments exist. It is an autosomal recessive disease caused by mutations in *ABCA4.* To date, a variety of gene supplementation approaches have been tested to create a therapy, with some reaching clinical trials. New technologies, such as CRISPR-Cas based editing systems, provide an exciting frontier for addressing genetic disease by allowing targeted DNA or RNA base editing of pathogenic mutations. *ABCA4* has ∼1,200 known pathogenic mutations, of which ∼63% are transition mutations amenable to this editing technology. In this report, we screened the known “pathogenic” and “likely pathogenic” mutations in *ABCA4* from available data in gnomAD, Leiden Open Variation Database (LOVD), and ClinVar for potential PAM sites of relevant base editors, including *Streptococcus pyogenes* Cas (SpCas), *Staphylococcus aureus* Cas (SaCas), and the KKH variant of SaCas (Sa-KKH). Overall, of the mutations screened, 53% (ClinVar), 71% (LOVD), and 71% (gnomAD), were editable, pathogenic transition mutations, of which 35–47% had “ideal” PAM sites. Of these mutations, 16–20% occur within a range of multiple PAM sites, enabling a variety of editing strategies. Further, in relevant patient data looking at three cohorts from Germany, Denmark, and China, we find that 44–76% of patients, depending on the presence of complex alleles, have at least one transition mutation with a nearby SaCas, SpCas, or Sa-KKH PAM site, which would allow for potential DNA base editing as a treatment strategy. Given the complexity of the genetic landscape of Stargardt, these findings provide a clearer understanding of the potential for DNA base editing approaches to be applied as *ABCA4* gene therapy strategies.

## Introduction

Stargardt macular degeneration (STGD1) is the most common inherited childhood blindness worldwide, with a prevalence of 1 in 8–10,000 (Cremers et al., 2020; Piotter et al., 2021). Furthermore, potentially pathogenic *ABCA4* alleles have a population frequency of 1:20, underscoring the impact of *ABCA4* in retinopathies (Maugeri et al., 1999; Yatsenko et al., 2001; Jaakson et al., 2003). Most individuals first experience symptoms at a young age, and become severely visually impaired or legally blind by their 4th–7th decade of life. The most common form, Stargardt 1 (STGD1), is a recessively inherited retinal degenerative disease occurring due to mutations in the *ABCA4* gene*.* ATP binding cassette protein family member 4 (ABCA4) is a transport protein critical in the visual cycle and therefore important for maintaining retinal health and function (Beharry et al., 2004; Quazi et al., 2012). Specifically, ABCA4 is located in the photoreceptor outer segments where the protein moves retinoids from the cytoplasm to the lumen *via* a flippase mechanism in both rod and cone photoreceptors (Zhang et al., 2015). Mutations in the *ABCA4* gene have a variety of outcomes on protein function, leading to misfolding and reduced function or loss-of-function, and therefore negatively affecting the visual cycle. This typically results in the build-up of retinoids which form bis-retinoid fusion products (Sparrow et al., 2010). Over time, as the retinal pigment epithelial cells (RPE cells) phagocytose the photoreceptor outer discs, the bisretinoids then build-up in the RPE, eventually causing cell damage and degeneration. As photoreceptor survival depends on the RPE cells, the photoreceptor cells undergo damage, and degeneration subsequent to the RPE, causing loss of central vision that progresses over time (Molday and Zhang, 2010). STGD1 has many phenotypic presentations, often with little genotypic correlation, making diagnosis a difficult process. For example, it has been shown that some missense mutations cause a more severe phenotype than some truncated proteins (Zhang et al., 2015). Alongside this, there are ∼1,200 known pathogenic variants, creating a complex genetic landscape (Allikmets, 2007; Cornelis et al., 2017; Cremers et al., 2020). Given the slow progression of the disease, there exists an ample treatment window, however, no treatments currently exist although various forms of therapy have been investigated (Cremers et al., 2020; Piotter et al., 2021).

To date, much of the research into STGD1 treatment options has focussed on either small molecules targeting various points in the visual cycle or gene supplementation therapy. However, gene supplementation therapy, while providing great potential in the realm of inherited retinal degenerative diseases, has faced many difficulties for STGD1 due to the large size of the *ABCA4* coding sequence of 6.8 kb. The preferred gene therapy delivery system, adeno-associated viral (AAV) vector, only has a carrying capacity of ∼4.7 kb (Grieger and Samulski, 2005). To overcome this, multiple alternative approaches have been tested, such as a dual vector approach (Trapani et al., 2014; Trapani et al., 2015; McClements et al., 2019), lentiviral vector delivery (Binley et al., 2013), nanoparticles (Sun et al., 2021), and intein-mediated reconstitution (Tornabene et al., 2019). These studies have had varying degrees of success (more details can be found in detailed reviews, Cremers et al. and Piotter et al.) (Cremers et al., 2020; Piotter et al., 2021). While any of these approaches would be immensely beneficial as a treatment option and would offer a single treatment option regardless of the *ABCA4* mutation, it is unknown for how long transgenes express, and show improvements in humans, given that retinal degenerations often progress over a lifetime (Cideciyan et al., 2013; Bainbridge et al., 2015; Jacobson et al., 2015; Gardiner et al., 2020; Suh et al., 2021). However, the most recent follow-up results from the Voretigene Neparvovec (Luxturna) Phase III clinical trial indicate continued improvements after 4-years (Maguire et al., 2021).

**FIGURE 1:**
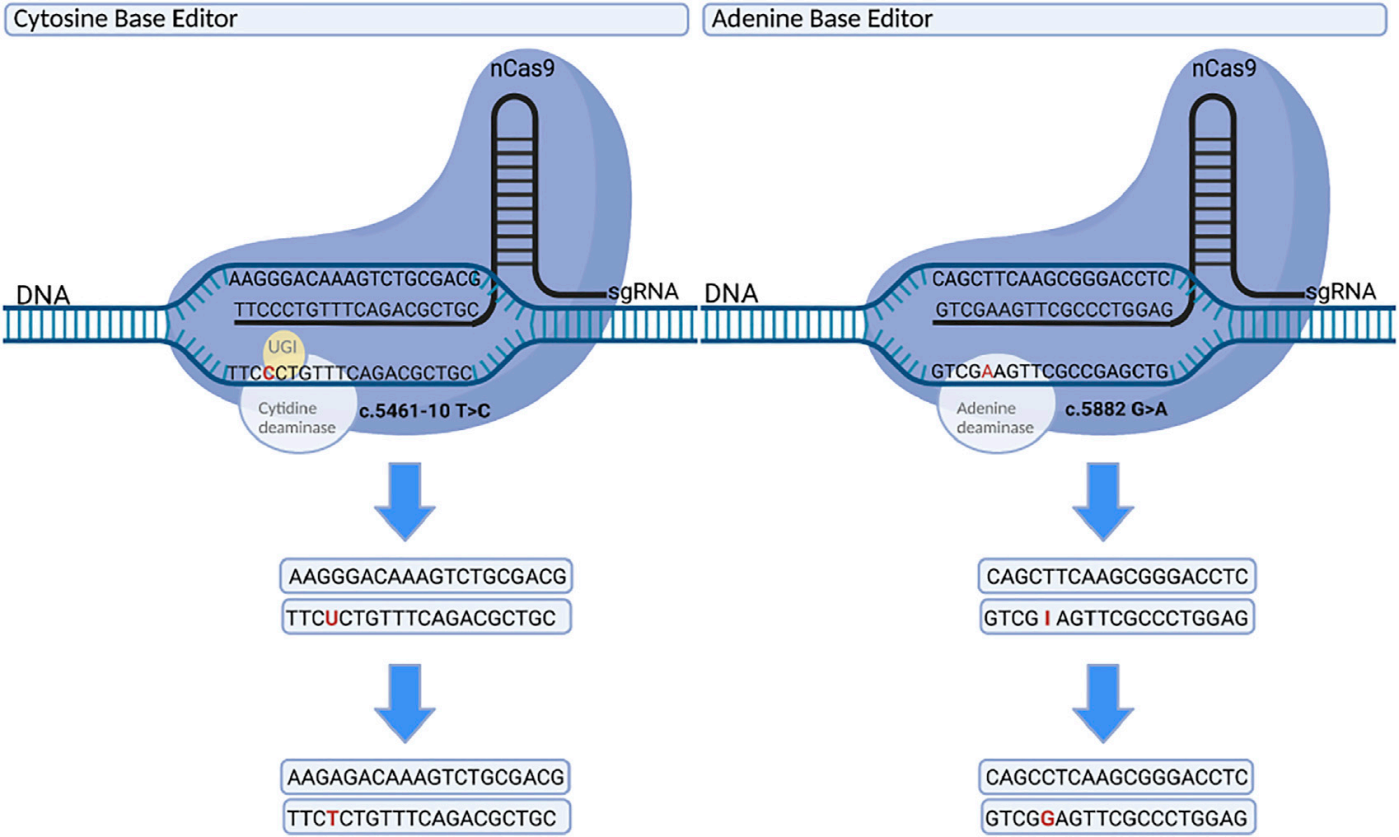
Cytosine base editor (left) and adenine base editor (right) showing respective single base corrections of common ABCA4 mutations, c.5461-10T > C and c.5882G > A. Cas9 complexes with a guide, interrogates the genome for the matching sequence, and binds. The opposite strand is freed, allowing the deaminase domain to act on the target base. This mediates C > T and A > G changes, enabling correction of all four transition mutations: G > A, A > G, C > T, and T > C. Created with BioRender.com.

Recent advances in gene editing using Clustered Regularly Interspaced Short Palindromic Repeats—CRISPR Associated Systems (CRISPR-Cas), have allowed for the development of a wide range of precision editing tools. Of particular interest for a mutation-rich gene, such as *ABCA4*, are the base editing systems adenine base editors (ABEs), and cytosine base editors (CBEs). These systems consist of a deactivated, or dead, Cas (dCas) fused to a deaminase domain (Gaudelli et al., 2017). In ABEs, a guide RNA leads dCas is used to find the genomic target, upon which the deoxyadenosine deaminase (TadA domain) can mediate the catalysis from adenine (A) to inosine. Inosine is functionally read as guanosine, thereby enabling adenine to guanine (G) editing. Likewise, CBEs use a guide lead dCas domain to locate the target, where the cytidine deaminase (APOBEC domain) can then deaminate the target cytosine (C) to uracil (U), mediating C to T editing (Kantor et al., 2020; Huang et al., 2021). Combined, these systems enable editing of all four transition mutations: G > A, A > G, C > T, and T > C (Figure 1).

In a recent study investigating all Leiden Open Variation Database (LOVD) entries of *ABCA4,* these four transition mutations made up 63% of all pathogenic mutations (Fry et al., 2021). Given the high number of pathogenic mutations found in *ABCA4* and the high frequency of heterogeneity, base editing may provide a treatment solution. However, there are a number of shortcomings to consider. Not only will each mutation require a unique guide sequence, logistically, one of the predominant issues often arises in the limitations of relevant protospacer adjacent motif (PAM) sites near the mutation. PAM sites are species-specific sequences which the dCas uses alongside the guide to identify an editing target. For a mutation to be targetable, there must be a nearby PAM for the dCas9 to find. The most common and effective Cas systems to date (in editing efficiencies) are *Streptococcus pyogenes* Cas9 (SpCas), *Staphylococcus aureus* (SaCas), and *Staphylococcus aureus*-KKH (SaKKH). These three Cas species offer PAM versatility and have verified base editing potential (Ran et al., 2015; Editas Medicine, 2021; Suh et al., 2021; Villiger et al., 2021).

To identify the Cas base editing targeting potential of mutations in *ABCA4*, we have investigated the pathogenic entries for *ABCA4* in the Genome Aggregate Database (gnomAD) v2.1.1, the Leiden Open Variation Database, and ClinVar, alongside patient data from three patient cohorts from Germany (Birtel et al., 2018), China (Jiang et al., 2016), and Denmark (Duno et al., 2012). The data were screened first for variant and mutation type, followed by screening of relevant transition mutations for nearby PAM sites. Specifically, we looked at SpCas, SaCas, and SaKKH PAM sites, as these are the most verified constructs to date and, therefore, most relevant for translation to clinical work at this time. Further, SaCas and SaKKH have a gene size of ∼3.2 kb, enabling packaging in AAV for gene therapy delivery individually (Friedland et al., 2015). However, paired with other necessary base editing components (TadA, APOBEC, etc.), the constructs are often too large to fit in an AAV vector, requiring a dual delivery strategy. Here, we aim to provide a better understanding of the number of mutations which can realistically be targeted using base editing systems in the mutation rich landscape of *ABCA4*-related Stargardt disease. We show that most transition mutations have one of the three described PAM sites within the currently defined editing window, which translates to 36–46% of total investigated mutations. Further, cohort analysis shows 44–76% of patients having at least one PAM site, largely due to heterogeneity, enabling multiple potential editing strategies. Overall, base editing, despite existing logistical frameworks, shows great potential as a treatment for Stargardt.

## Methods

**FIGURE 2:**
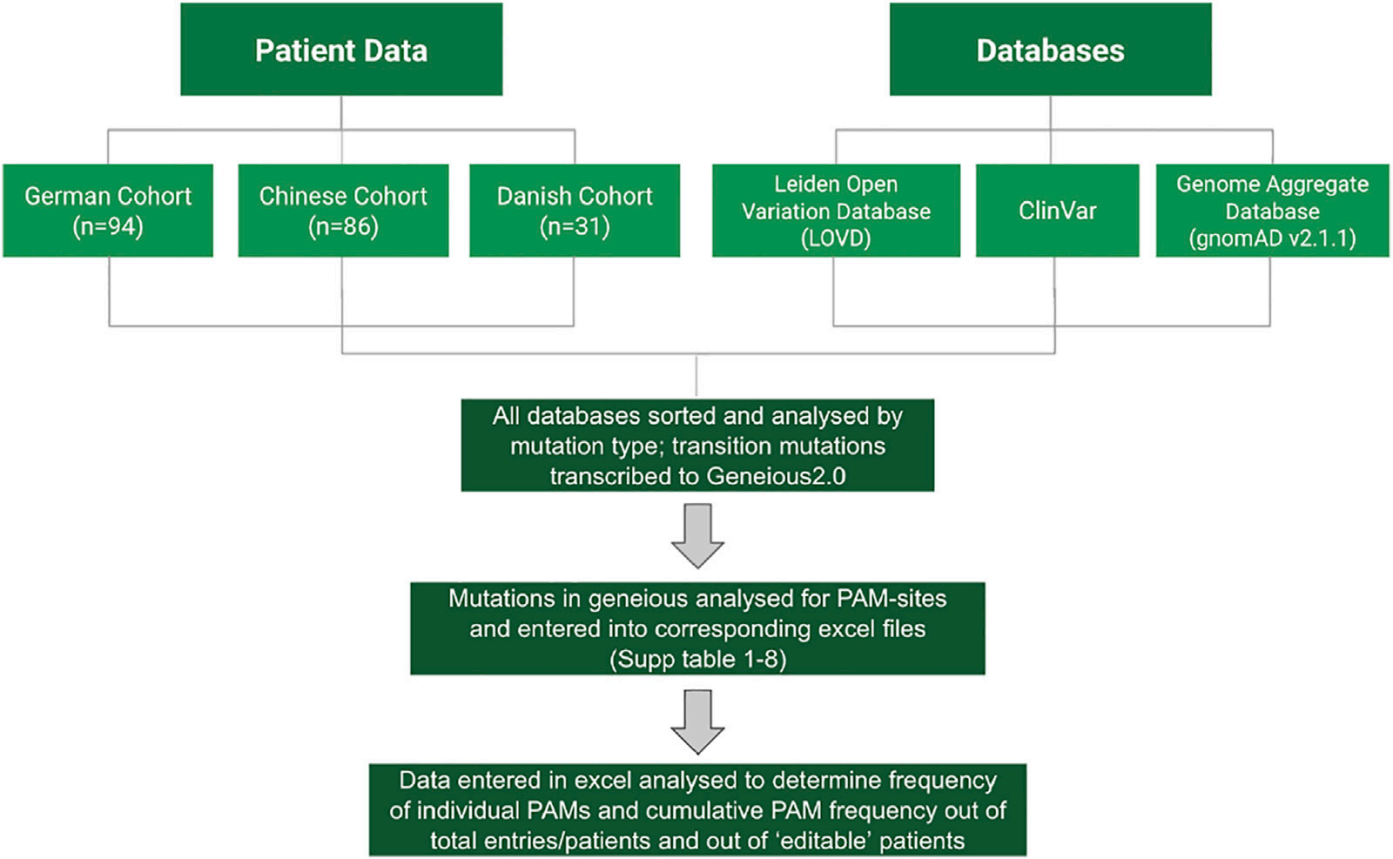
Flow chart of the different data used and the analysis process.

**All relevant data analyzed can be found in Supplementary Tables S1–S8. A flow chart of the methods is provided in Figure 2.

### gnomAD v2.1.1 Database

3,979 *ABCA4* variants were downloaded from the Genome Aggregation Database (gnomAD) v2.1.1 (https://gnomAD.broadinstitute.org/gene/ENSG00000198691?dataset=gnomAD_r2_1) on June 17th, 2021 (Karczewski et al., 2020). All variants were screened for transition mutations. They were then separated on the basis of the ClinVar classification. To provide analysis parameters, the mutations with the “pathogenic” and “likely pathogenic” ClinVar classification were extracted, totaling 205 mutations. These were then screened by mutation type (missense, nonsense, splice site, other) and base change (Supplementary Tables S5, S6). Lastly, those classified as “conflicting interpretations of pathogenicity,” were not included in the data but specific mutations were used as examples in the discussion.

### ClinVar Database

1,072 Stargardt variants were downloaded from ClinVar on Sept 12, 2021. All variants were initially screened to only include “*ABCA4*”as the causative gene, leaving 690 remaining variants. These were screened further, based on their clinical significance, for “pathogenic” and “likely pathogenic” variants. As in the gnomAD dataset, the “VUS,” “Conflicting interpretations of pathogenicity,” “uncertain significance,” “benign,” and “likely benign,” were excluded from analysis. The remaining dataset included 279 “pathogenic” and “likely pathogenic” variants. These were then screened for transition mutations and by mutation type to better characterize the dataset (Supplementary Tables S7, S8).

### LOVD Database

6,540 Leiden Open Variation Database v.3.0 pre-screened *ABCA4* entries were analyzed from a previously published source with 679 unique entries (Fry et al., 2021). Repeat entries were not deleted to compare database input. This aligns with the total 698 *ABCA4* entries in ClinVar, which were filtered to only include “confirmed” pathogenic STGD variants. We looked at all three datasets to account for the clear discrepancy seen between the datasets. LOVD entries were screened for editable versus non-editable mutation types, where transition mutations were screened for PAM sites and transversion mutations and indels were labeled “NA.” PAM sites were screened as in the other datasets, the method for which is described in greater detail below. All data can be found in Supplementary Table S1.

### PAM Site Screening and Cas Parameters

To search for PAM sites, the human *ABCA4* sequence (*ABCA4-24*) was downloaded from the National Center for Biotechnology Information (NCBI) database within Geneious Prime® 2020.2.4 (Geneious) (Gene ABCA4, 2004). All of the “pathogenic” and “likely pathogenic” mutations extracted from the gnomAD, ClinVar, and LOVD databases were manually annotated on the *ABCA4*—24 reference file and the surrounding area screened for PAM sites of three different Cas: *Streptococcus pyogenes* Cas9 (SpCas), *Staphylococcus aureus* Cas9 (SaCas), and the KKH variant of *Staphylococcus aureus* Cas9 (SaKKH)*.* The PAM sites screened were therefore 5′-NGG (SpCas), 5′-NNGRRT (SaCas), and 5′- NNNRRT (SaKKH). While SaCas has reported editing with the 5′-NNGRRV/N-3′ PAM, for this analysis the canonical 5′-NNGRRT-3′ PAM was used. For consistency, the guide length used was 20 base pairs from the 5′ end of the PAM site for all three variants (Note, SaCas can have effective guides up to 24 bp long) (Friedland et al., 2015). Given that only specific regions of the 20 base pair guides are likely to be targetable with base editors, parameters from past papers were incorporated to reflect this: for SpCas, the mutation had to fall between positions 4–8 (Porto et al., 2020), whereas for SaCas and SaKKH, at positions 4–12 (Porto et al., 2020), and positions 2–15 (Evanoff and Komor, 2019; Porto et al., 2020; Zhang et al., 2020a), respectively (position number 1 starts from the 5′ end of the guide). While Porto et al. (2020), describes the SaKKH editing window as 4–12, Zahng et al. 2020, describes editing from positions 1–15. Thus, the window described in Evanoff et al. 2019, of positions 2–15 was used for this analysis. Lastly, the type of base editor used (e.g., ABE8e *vs.* ABE7) affects the editing window for different mutation types (i.e., C > T *vs.* A > G) (Porto et al., 2020). For this analysis, the described editing windows were used regardless of mutation type as they are a tentative middle ground and the systems are continually evolving. In the case of an SaCas PAM (5′-NNNGRRT) with a mutation at positions 2–3 and 13–14, they were included with SaKKH. The results also show an “SaCas + SaKKH” column given how closely related these versions are. These were considered the “ideal” targeting windows but are seen as guidelines, given the extreme variability of editing at different target sites and the often seen high function of non-canonical PAM-sites. PAM sites which did not meet the parameter criteria but occurred within the guide length (for example, an SpCas PAM site with the mutation at position 3 or 17) were noted and accounted for separately in the data set. This included any PAM that would put the target within the 20 bp guide, to account for the variability observed (Molla and Yang, 2019; Zhang et al., 2020a). For example, many evolved SpCas variants show larger or shifted editing windows (Kantor et al., 2020; Porto et al., 2020). Lastly, bystander edit analysis for individual sites, however, this is a major consideration when targeting a mutation.

### Patient Data

Anonymised patient data were downloaded from three previously published inherited retinal degenerative disease studies describing cohorts from different countries: Germany, Denmark, and China (Duno et al., 2012; Birtel et al., 2018; Hu et al., 2019). The data were first sorted by gene to separate patients with no more than two different mutations in *ABCA4* i.e., complex alleles were annotated*.* Next, as with the gnomAD data, the remaining transition mutations were annotated using GeneiousPrime*.* The mutation location in relation to potential PAM sites were identified as described above. Mutations already in the gnomAD file were not annotated again, rather the PAM sites listed in the gomAD file were transcribed to the patient data file. Patient data can be found in Supplementary Table S2 (German “editable”), 2.1 (German-raw), 3 (Chinese), and 4 (Danish).

## Results

### Analysis of Targetable Mutations in the gnomAD v2.1.1, ClinVar, and LOVD Databases

**FIGURE 3:**
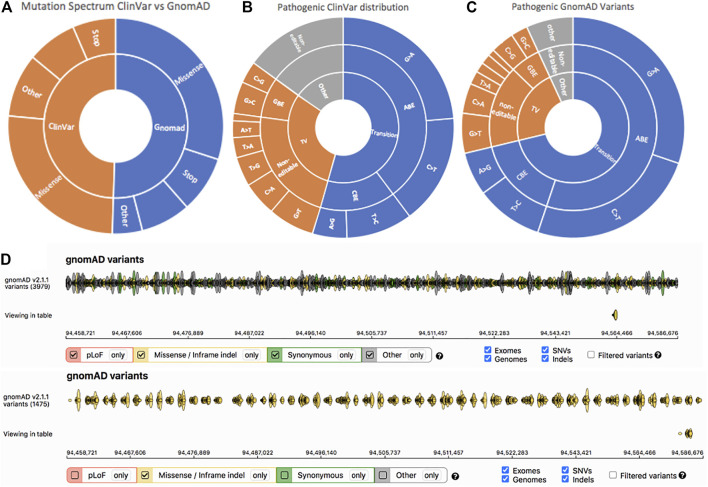
**A)** Charts showing distribution of mutation classification and type in both ClinVar and gnomAD. Missense mutations were the most prevalent overall in both ClinVar and gnomAD. making up 63% of mutations. Pathogenic mutations were then characterised for both ClinVar **(B)** and gnomAD **(C)** data sets. Transition mutations made up a larger percentage of “pathogenic” and “likely pathogenic” mutations in gnomAD (71%) than in ClinVar (54%). However, when looking at the complete data set of each, transition mutations made up 62% of the gnomAD database and 59% of the ClinVar database. **(D)** A heatmap taken from gnomAD showing all gnomAD variants (top) and all missense variants (bottom).

The Genome Aggregation Database (gnomAD) v2.1.1 spans 125,748 exomes and 15,708 whole genome sequences from unrelated individuals. *ABCA4* has a total of 3,979 gnomAD entries, of which 62% represented transition mutations (G > A, A > G, T > C, C > T) (Figure 3C). Mutation type distribution was similar between Clinvar and gnomAD (Figure 3A). Mutations in *ABCA4* did not appear to occur in “hotspots”, rather they were spread evenly across the gene (Figure 3D). Similarly, in ClinVar, 59% of the 690 *ABCA4* Stargardt entries were transition mutations (Figure 3B).

**FIGURE 4:**
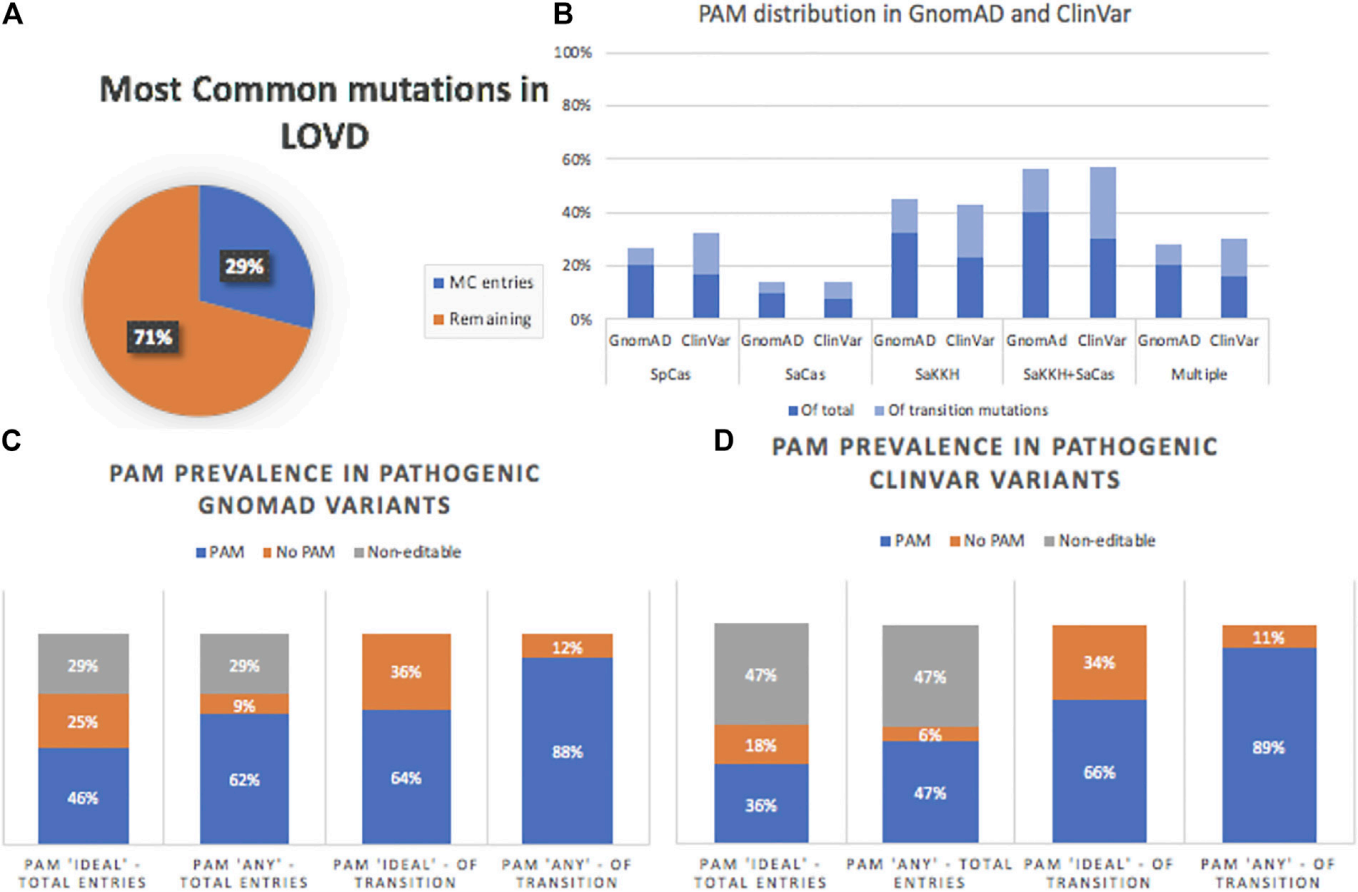
**A)** LOVD analysis was influenced by entry numbers, where 29% of entries consisted of the five most common (MC) mutations (c.5882G > A, c.5461-10T > C, c.2588G > C, c.3113C > T, and c.1622T > C. **(B)** Distribution of the various PAM-sites by database. Given the large editing window of SaKKH, this was the most prevalent PAM/Cas in both databases. The columns are additive—dark blue represents the percentage of total entries, whereas the percentage indicated by the light blue bar is taken out of the transition mutations. LOVD was excluded from this analysis as it was based on entry number. **(C)** and **(D)** PAM prevalence in gnomAD and ClinVar databases. “Ideal” PAM proximity accounted for 46 and 36%, respectively, of “pathogenic” and “likely pathogenic” mutations.

To analyse the base-editing potential of these transition variants, the mutations were analyzed in Geneious for nearby PAM sites. Each mutation was searched for relevant NGG (SpCas), NNGRRT (SaCas), and NNNRRT (SaKKH) PAM sites that would either enable mutation correction by targeting either the forward or the reverse strand, depending on the mutation. We found that in gnomAD and Clinvar, 64 and 66% of transition mutations had a nearby PAM site meeting all predetermined criteria, respectively (Figures 4C,D), with 28 and 30% having multiple “ideal” PAM options (Figure 4B). When taken in the context of all pathogenic mutations, this made up 46 and 36% of mutations (Figure c and d). Non-optimal PAM-sites were identified for which the mutation site was outside of the current predicted editing window. If included in the data set, this increased the total editable mutations to 88 and 89% of transition mutations i.e., 62 and 46% overall (Figures 4C,D). Conversely, only 25 and 18% transition mutations had no “ideal” PAM sites nearby.

Overall, in gnomAD and ClinVar, of the PAM-sites which met all the criteria, SaKKH had the highest prevalence, with ∼44% of transition mutations occurring near an SaKKH PAM-site. This was significantly higher than the ∼30% observed for SpCas, likely due to the significantly larger editing window. SaCas had the lowest prevalence, with ∼14% of transition mutations having a nearby SaCas PAM, given the more stringent PAM-site requirement alongside a narrower editing window. However, when combined, SaCas and SaKKH cover ∼56% of transition mutations, or 40% (gnomAD), and 30.5% (ClinVar) of all pathogenic mutations.

Given that the LOVD database is based on individual entries (i.e., multiple entries for the same variant), it was analyzed separately. Most notably, 29% of the entries consisted of the five most common mutations (Figure 4A). However, the distribution of transition mutations remained nearly constant regardless of whether these were included or excluded. Of total entries, 47% had an “ideal” PAM, which consisted of 66% of transition mutations. Further, when expanded to include “nearby,” non-optimal PAMs, this drastically increased to 67% overall and 95% of transition mutations.

### Patient Data

Patient data from three previously published cohorts were analyzed from: Germany (Birtel et al., 2018), Denmark (Duno et al., 2012), and China (Hu et al., 2019). Each dataset contains slightly different information, so the results gleaned varied. Overall, we found that 84% of the German cohort, 90.3% of the Danish cohort, and 84% of the Chinese cohort had editable transition variants. This reflects the findings of Fry et al. and Stone et al. looking at patient cohorts in Oxford and the United States, respectively, where 88.8 and 92.7% of *ABCA4* patients had editable transition variants (Stone et al., 2017; Fry et al., 2021). The three aforementioned cohorts were interrogated for relevant PAM-sites to gain a greater understanding of translatability at this point in the CRISPR journey.

### Patient Data—Bonn, Germany

**FIGURE 5:**
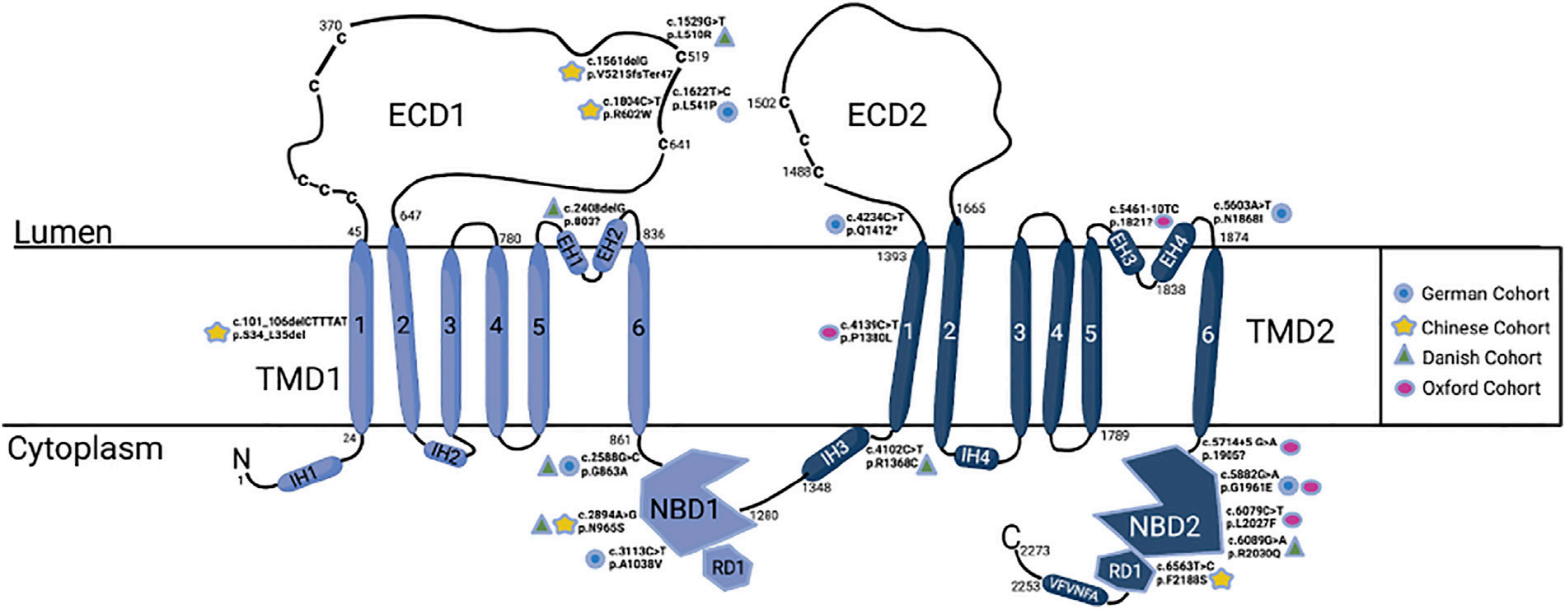
ABCA4 molecular structure (Liu et al., 2021a) with the five most mutations from each cohort. There are 3–4 notable hotspots: ECD1, NBD1, pre-TMD2, and NBD2. ECD, extracellular domain; NBD, nuclear binding domain; TMD, transmembrane domain. Blue circle- German cohort, yellow star- Chinese cohort, green triangle- Danish cohort, pink circle- Oxford cohort. Created with BioRender.com.

Patient data were extracted from a published study investigating 251 patients with cone-rod dystrophies (Birtel et al., 2018). Only patients with mutations in *ABCA4* were analysed from this data set, totalling 94 patients and 229 variants. This dataset had a wide range of mutation distribution, where only 4.2% of patients were homozygous for a single mutation, but 38% had complex alleles. Within this cohort of patients, 75.5% of total variants identified were missense changes with the remaining split relatively evenly between stop, splice, and “other,” at 9.8, 7.7, and 6.8%, respectively. 96.8% carried at least one missense change, with stop, splice, and other mutations occurring in 23.4, 19.1, and 17% of patients, respectively. Overall, 84% of patients had at least one targetable allele. However, only patients with compound transition mutations were deemed “editable” and made up 48% of the cohort. Lastly, the 5 most common mutations made up 43% of mutations, the distribution of which can be seen in comparison to other patient cohorts in Table 1, and locations of which can be found in Figure 5.

**TABLE 1:** Shows the top 5 mutations in each patient cohort and their prevalence. The bottom row shows combined prevalence within the cohort out of all mutations present (not patients). The proportion of PAMs represented in any cohort was affected by the type of most common mutations. For example, the German cohort had an unnaturally high prevalence of SaCas PAM sites due to c.5882 G > A mutation. The underlined mutations are seen in more than one cohort. The Oxford cohort data was taken from Fry et al.

German cohort Birtel et al. (2018)		Chinese cohort Hu et al. (2019)		Danish cohort Duno et al. (2012)		Oxford cohort Fry et al. (2021)	
c.5603A > T	14%	c.101_106	7.2%	c.2588G > C	11%	c.5882G > A	9.3%
		delCTTTAT					
c.5882G > A	10%	c.2894A > G	4.2%	c.2894A > G	6.5%	c.5461-10T > C	6.5%
c.1622T > C	6%	c.1804C > T	2.4%	c.1529G > T	6.5%	c.6079C > T	6.5%
c.3113C > T							
c.2588 G > C	5%	c.1561delG	2.4%	c.6089G > A	5%	c.4139C > T	5.1%
c.4234 C > T	2.6%	c.6563T > C	2.4%	c.4102C > T/c.2408delG	5%	c.5714+5G > A	4.7%
Total	43%	Total	18.6%	Total	34%	Total	32.2%

**FIGURE 6:**
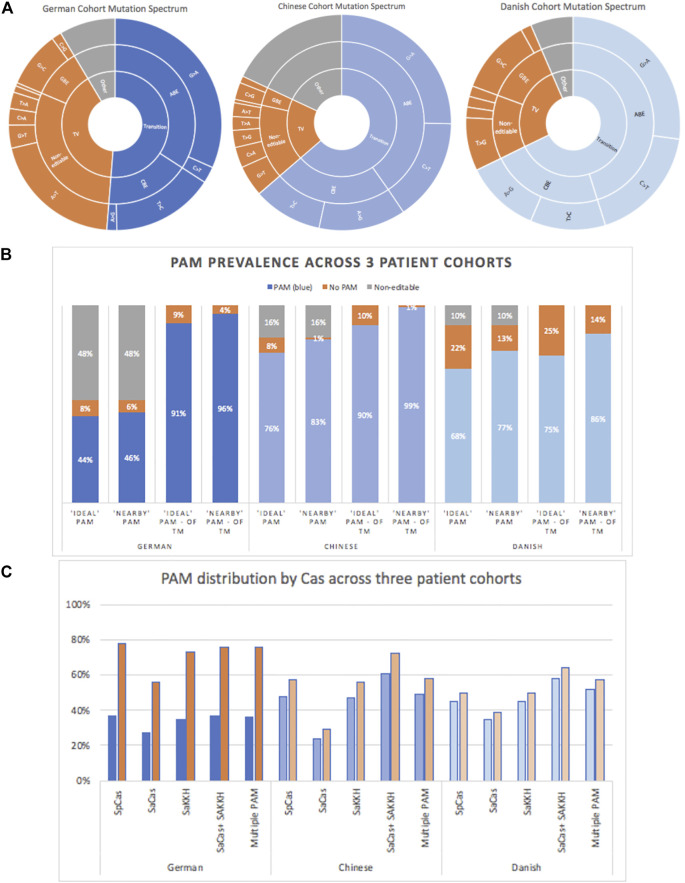
**A)** Mutation spectrums of patient cohorts in three countries. Transition mutations are typically the most prevalent. **(B)** PAM prevalence across German, Danish, and Chinese cohorts. “Ideal” and “nearby” PAMs are described in the methods. A cohort’s overall editability was greatly affected by mutation type e.g., complex alleles made-up 38% of the German cohort and were therefore not deemed “editable.” The vast majority of transition mutations, however, are editable. **(C)** PAM distribution by Cas species across the three patient cohorts. Was taken in the context of all patients in the cohort (total patients) and in the context of “editable” mutations i.e., compound, transition mutations. Blue = of total patients, orange = of patients with editable transition mutations.

For PAM-site analysis, patients with complex alleles were excluded from the data-pool. These were deemed non-targetable without further information regarding which mutations occur on which allele, as alleles were not specified. With this cohort refinement, 44% of all patients carried editable transition mutations within range of a nearby PAM, the correction of which may have a therapeutic outcome. Similar to the gnomAD and ClinVar database assessments, SaKKH PAM sites occurred with the highest prevalence, with 73% of targetable mutations. SaCas PAM sites had a much higher prevalence among mutations in this patient cohort at 56%, compared to the ∼14% in gnomAD and ClinVar listed data sets. This is likely due to the high prevalence of common mutations in a clinical cohort, such as c.5882 G > A, which was present in 19.8% of the “editable” patients and has both an SaKKH and SaCas PAM site. c.5882G > A has a population frequency of 0.4% in Europe and would therefore be expected to have a high prevalence in a German cohort (Cremers et al., 2020). Interestingly, c.5882G>A was not seen in the Danish cohort. SpCas PAM sites were also prevalent in a range of mutations in this patient cohort, with 78% of patients having a targetable mutation. Overall, 91% of editable mutations had an “ideal” PAM, or 44% of the cohort, with 76% having more than one PAM site, enabling multiple editing strategies, and providing increased chances of success (Figures 6B,C).

Given the high rate of complex alleles (38%), the German cohort would be particularly amenable to multiplex editing, where multiple guides are provided to one base editor to enable correction of multiple mutations simultaneously. For ABEs, this would require G > A/G > A, G > A/C > T, or C > T/C > T mutations, and CBEs would require A > G/A > G, A > G/T > C, or T > C/T > C. Of the patients with 3 or more mutations, 52% had a mutation type combination conducive to multiplexing. However, mutation location was not disclosed and would affect the possibility of a multiplexing approach. Furthermore, PAM sites were not investigated for these patients, which would have to be considered for this approach.

### Patient Data—Chinese Cohort

Patient data were taken from a published Chinese cohort consisting of 86 *ABCA4* Stargardt patients, three of which had complex alleles and 8 that were homozygous (Hu et al., 2019). When both including and excluding the patients with complex alleles, 81% had at least one transition mutation (This number stayed the same as all three had a transition mutation, so were removed from the total “editable” pool, as well). Overall, the mutation spectrum including all patients comprised 29.3% G > A, 15% A > G, 18% C > T, and 12% T > C, totaling at 70% (Figure 6A). G > C and C > G only consisted of 4% of mutations. The five most common mutations in this cohort consisted of nearly 19% of mutations overall, as seen in Table 1. One of these, c.2894A > G, is a known founder mutation in the Chinese population (Jiang et al., 2016). Interestingly, this is also a founder mutation in the Danish population (Rosenberg et al., 2007).

Of the total patients, 76% had at least one PAM-site on one allele. However, of patients with editable transition mutations, this increased to 90% having at least one PAM-site (Figure 6B). Conversely, of total patients, only 8% did not have a transition mutation with a nearby PAM-site. The PAM distribution was different in the Chinese cohort compared to the German cohort, likely due to a greater mutation diversity. Of patients with transition mutations, 57% had an SpCas PAM, closely followed by SaKKH, with 54%. SaCas occurred significantly less, at only 29% (Figure 6C). However, despite the relatively low number of SaCas, it is significantly higher than in the databases. This is likely due to the high presence of heterogeneity in the patient cohorts, and thus a greater opportunity for a PAM site to be present.

### Patient Data—Danish Cohort

Danish cohort data were taken from a published cohort that included 31 *ABCA4*-related retinopathy patients (Duno et al., 2012), of which three had complex alleles and none were homozygous. 88% of total patients had at least one transition mutation. The prevalence by mutation type of total mutations consisted of 27% G > A, 11% A > G, 18% C > T, and 11% T > C (Figure 6A). Of these patients with transition mutations, 77% had at least one PAM-site, accounting for 68% of patients overall. Half of patients with transition mutations had more than one PAM-site, enabling multiple editing strategies. The five most common mutations in this cohort accounted for 34% of mutations present. The most common mutation, c.2588G > C, shows conflicting pathogenicity dependent on other mutations, but is often described as mild (Cremers et al., 2020). The second most common mutation, c.2894A > G, is a known founder mutation in the Danish and Chinese populations, and has multiple PAM options(Cremers et al., 2020).

SpCas and SaKKH PAM sites near mutations were equally represented in the Danish cohort, both at 45% of the total cohort, whereas SaCas was present for 35% of patients. 52% of patients had multiple PAM sites (Figures 6B,C). The percentages only increased slightly when taken from patients with transition mutations (“editable”), because only three patients did not have at least one transition mutation. SpCas and SaKKH increased slightly to 50%, whereas SaCas increased to 39%. Similar to the German and Chinese cohort, the highly heterogeneous Danish cohort had a high level of SaCas PAM sites, in particular, relative to gnomAD, and ClinVar.

## Discussion

In Gaudelli et al., it was shown that in the human genome, of roughly 32,000 pathogenic point mutations, 62% were transition mutations, and thus theoretically editable using ABEs or CBEs (Gaudelli et al., 2017). Another study, Xu et al., looked at 53,469 human pathogenic mutations, where 42.8% could be potential targets for base editing (Xu et al., 2021a). Mutations in *ABCA4* follow this trend, with 63% of LOVD entries being theoretically editable transition mutations (Cremers et al., 2020; Fry et al., 2021). When looking at the other available *ABCA4* databases, we found that 62% of gnomAD entries were transition mutations, reflecting prior findings, while only 59% of Stargardt-*ABCA4* mutations in ClinVar were transition mutations. When screening only pathogenic mutations in gnomAD the number increased to 71.2%, whereas in ClinVar it decreased to 53%. This reflects the greater number of “other” mutations (indels, duplications, etc.) in the pathogenic ClinVar dataset, at 19%, compared to only 9% in gnomAD. Despite these variabilities, across all databases, *ABCA4* consistently has a prevalence of transition mutations consistent with the rest of the human genome. Thus, DNA base editors provide an exciting opportunity to correct this large proportion of pathogenic mutations and therefore provide therapeutic benefits for Stargardt and other *ABCA4-*retinopathy patients.

Base editors have shown *in vivo* activity in correcting a multitude of pathogenic mutations, including 29% editing efficiency in *RPE65* via lentiviral delivery in photoreceptors (Yeh et al., 2020; Koblan et al., 2021; Suh et al., 2021; Xu et al., 2021a). Efforts are ongoing in finding effective delivery methods for larger DNA base editing constructs, such as via dual AAV vector (Hung et al., 2016; Villiger et al., 2018; Levy et al., 2020; Yeh et al., 2020), lentiviral vector (Suh et al., 2021), and nanoparticle delivery (Zuris et al., 2015; Zhang et al., 2017; Wei et al., 2020). Furthermore, DNA base editing constructs have rapidly evolved, expanding PAM site possibilities and reducing construct size (Molla and Yang, 2019; Kantor et al., 2020; Porto et al., 2020). While this describes a bright future for base editors, many new constructs have only been minimally optimized and verified, and not tested *in vivo*, and thus have limited therapeutic potential at this time. In addition, gaining a strong understanding of off-target editing, bystander effects, and toxicity are key for these constructs in providing therapeutic potential. This paper aimed to shed light on “editable” transition mutations on the basis of nearby PAM sites for the currently most verified and therapeutically relevant Cas species—SpCas, SaCas, and SaKKH. By analyzing “pathogenic” and “likely pathogenic” variants in different databases, we have established a baseline for how many transition mutations are, in fact, editable by prevalent base editing systems.

In ClinVar and gnomAD, transition mutations made up a large proportion of the editable mutations, at 59 and 63%. As mentioned above, Xu et al. found that 42.8% of 53,469 human pathogenic mutations in ClinVar had base editing potential. Of these, 72.4% were *not* amenable to SpCas base editing due to the 5′-NGG PAM limitation (Xu et al., 2021a). Similarly, when looking at variant databases, we found that ∼70% did not have SpCas PAM sites nearby. But, when looking at the three Cas combined, we found that ∼65% of transition variants had a PAM site. Further, when looking at patient data, PAM prevalence increased significantly due to the majority of patients being heterogeneous and often having at least one transition mutation; the German, Chinese, and Danish cohorts showed transition mutations in 84, 84, and 90% of patients, respectively. PAM prevalence of “ideal” PAM sites of the total patient cohorts (in the same order German, Chinese, and Danish) was 44, 76, and 68%, which, when taken of total transition mutations (as in Xu et al.), increased to 91, 90, and 75%.

In a recessive condition such as Stargardt disease, being able to correct one pathogenic mutation would be anticipated to provide therapeutic benefit to a large proportion of patients. Targeting prominent founder mutations and common pathogenic mutations would address a great need within any given population. In the German and Oxford cohorts, c.5882G > A accounts for roughly 10% of all mutations present and has three different “ideal” PAM opportunities. Moreover, most of the occurrences (18 of 22) arose in individuals with compound alleles, meaning correction of the mutation may ameliorate disease. This founder mutation originated in East Africa, and is thus also seen frequently in Somalia, Kenya, and Ethiopia (Burke et al., 2012; Cremers et al., 2020). In the Chinese cohort, due to mutation type, more than half of the most common mutations are not amenable to base editing. Likewise, only one-third of the most common mutations in the Danish cohort are amenable to base editing due to base change and PAM availability. Interestingly, the second-most common mutation in both cohorts [c.2894A > G; p.N965S], is reported to be a founder mutation in both China and Denmark, and has multiple “ideal” PAM sites (Rosenberg et al., 2007; Jiang et al., 2016; Cremers et al., 2020; Zhu et al., 2021). While the most common mutations in the Danish and Chinese cohorts, c.2588 G > C and c.101_106delCTTTAT, are not amenable to editing by ABE or CBE, new advances in CRISPR-Cas, such as prime editing, and glycosylase base editors (GBEs) will likely enable editing in the future.

Prime editing (PE) enables all 12 transition and transversion edits and correction of small insertions and deletions. The most recent generation, PE3, shows high on-target editing efficiency of up to 33%, with reduced off-target editing (Anzalone et al., 2019; Kantor et al., 2020). In addition, a recent study optimized PE2 and tested it *in vivo via* dual-AAV split intein delivery to correct a pathogenic mutation. While the editing rate was too low for there to be a therapeutic effect for this disease, low rates of editing were still seen (Liu et al., 2021b). Similarly, GBEs, which correct C > G and G > C mutations in mammalian cells, show high rates of on-target specificity at position 6, with editing efficiencies ranging from 5.3 to 53%, but with a strong preference for position 6 within the guide (Zhao et al., 2021). While both of these methods show great promise, they are significantly less verified *in vivo,* thus requiring more optimization and study.

Other targeting challenges became apparent in the German cohort, where 38% of patients had at least one complex allele. This is higher than the 10% mentioned in Cremers et al. (Shroyer et al., 2001; Zhang et al., 2015; Cremers et al., 2020) While this could be affected by dataset size, this is likely also affected by it being a German cohort. Germany has a deleterious founder mutation which is a complex allele, c [1622T > C; 3113C > T], p [Leu541Pro; Ala1038Val], which constitutes 34% of all complex alleles (Cornelis et al., 2017; Cremers et al., 2020; Rivera et al., 2000). This aligned closely with the 36% found in this cohort. The complex allele [c.2588G > C, c.5603A > T] arose in 12% of patients in the cohort overall, and consisted of 30% of patients with complex alleles, lower than the ∼50% described in Cremers et al. The high prevalence of complex alleles, however, brought down the number of patients with editable transition mutations to 48%. Of these, 91% had “ideal” PAM sites nearby. In comparison, the Danish cohort only had 6.5% complex alleles, both of which were the c [1622 T > C; 3113 C > T], p [Leu541Pro; Ala1038Val] German founder mutation. The Chinese cohort was noted to include patients with two or three mutations (but not more), yielding 3.5% with complex alleles. While the latter two cohorts show low rates of complex alleles, the datasets are either significantly smaller or actively aimed to exclude complex alleles of more than three mutations.

Depending on the distribution of complex alleles, patients with more than two mutations could have targetable mutations where, if edited, a therapeutic effect may be seen (i.e., if a patient with 3 mutations has two on one allele, and 1 on the other). In addition, if multiple mutations have the same PAM site and are amenable to the same base editor, guides could be multiplexed to target multiple mutations simultaneously (McCarty et al., 2020). In Kurata et al., it was shown that Cas9-knockdown multiplexing using 10 gRNAs targeting 10 different loci effectively edits some of the targets—the first three guides showed the highest rates of knockdown, with waning efficacy thereafter (Kurata et al., 2018). Recently, multiplexing was also shown to work well using an SaKKH-CBE alongside an ABE in cynomolgus monkey embryos targeting *EMX1*, *FANCF*, and *BRCA1*. Five of eight embryos were edited at all three sites, with editing efficiencies of 47–100% for C > T conversions and 10–86% for A > G conversions (Zhang et al., 2020b). Although promising, this is best viewed as a proof-of-principle, given that delivering two base editors is highly unlikely, currently. Nonetheless, given the high rates of complex alleles in European populations, and ∼10% overall, multiplexing to target mutations using the same base editor would enable a treatment potential for a greater patient base.

Opposite to complex alleles, *ABCA4* has been reported to have high rates of monoallelic and “no mutation” clinically confirmed cases of *ABCA4-*disease. Specifically, one study found that 20–25% of cases are monoallelic and 10–15% have no mutation (Zernant et al., 2017). Interestingly, in the German cohort, five patients (not included in the 94 analyzed patients) had only one mutation in *ABCA4* but displayed phenotypes of *ABCA4-*associated disease, accounting for 5.5% of *ABCA4* patients. Two of these patients had common mutations (c.5882G > A, c.1622T > C; c.3113C > T), while two had novel mutations (Birtel et al., 2018).

Apart from therapeutic applications, base editors can be applied in gaining a greater understanding of the effects of individual variants, particularly in complex alleles, given the immense number of mutations, and their varying roles individually versus in relation to other mutations. Specifically, known mutations in *ABCA4* result in various phenotypes/pathogenicity depending on whether in *cis* or *trans* of another mutation. For example, c.2588G > C is only causal in *cis* to c.5603A > T (Zernant et al., 2017; Cremers et al., 2020).

Fortunately, across the analyses, we found that the majority of transition variants had at least one of the desired nearby PAM sites, especially in the patient data. While the extreme heterogeneity observed often corresponded to complex alleles, in compound heterozygous patients, it would enable a greater number of editing approaches. Of German compound heterozygous patients, 91% had a relevant PAM site nearby, of which 76% had more than one. Likewise, the Danish and Chinese cohort showed that, of the cohorts overall, 68 and 76%, respectively, had “ideal” PAMs. Roughly half of these had more than one PAM. Having multiple PAM options per patient provides flexibility when designing editing strategies in terms of the Cas system used and guide design.

In this paper, a 20 bp guide length was chosen as a parameter, however, guides of varying lengths have worked successfully (Friedland et al., 2015). Further, Suh et al. recently demonstrated in a proof-of-principle *in vivo* study targeting *RPE65* with an SpCas ABE using a lentiviral delivery system, that ABEs work in the retina and that the PAM sites were more versatile than anticipated*.* Indeed, higher editing rates of 29% were observed using SpCas9 targeting previously identified non-canonical PAM sites (NAG and NGA) (Hsu et al., 2013; Jiang et al., 2013; Zhang et al., 2014), than with the flexible xCas9 (3.7) (Suh et al., 2021). This increased PAM flexibility would likely expand the number of transition mutations amenable to base editing extensively amongst all databases and patient cohorts. In addition, the target window is also flexible. Although a larger target window enables greater editing potential, it also increases the likelihood of introducing an unintended bystander edit due to the increased likelihood of the same base occurring within a larger editing window (e.g., if targeting an A, that there is another nearby A). This may be particularly problematic in *ABCA4*), where there are ∼1200 known pathogenic variants, and thus bystander edits could more likely be detrimental. Where SaKKH had the biggest target window of the investigated Cas’ and therefore often targeted as many variants as SpCas, this may inversely also be less safe therapeutically, given the greater potential for bystander edits. However, although the parameters outlined in previous studies provide strong guidance for guide design and targeting, individual target sites require testing of multiple guide designs to see which works best. For *ABCA4*, with ∼1200 pathogenic mutations, this may be a limitation. However, some mutations are far more common than others and would address a significant portion of the patient population. For example c.5882G>A is seen in ∼10% of European patients. In addition, while SpCas works demonstrably well *in vitro* and *in vivo*, a present constraint is delivery into the cell, due to its large size.

The data we present in this study provides great insight into the therapeutic potential of base editors for the treatment of Stargardt disease, but with some limitations. First, the vast majority of the gnomAD entries, 92.6%, were unclassified and thus not used in our analysis. Manually screening these and performing literature reviews or cross-referencing with other databases would provide insight into these unlabeled entries and provide significantly more data points. Second, common mutations, such as c.5882G > A, were typically categorized as “conflicting interpretations of pathogenicity,” and were therefore excluded from the databases due to the ambiguities. However, these were partially accounted for in the patient datasets. Additionally, the gnomAD and ClinVar datasets do not take into account variant prevalence differences across populations (They are indicated in gnomAD, but these were not used in this analysis, as many of the listed pathogenic entries have an allele count of “1.”) The patient data from different countries aimed to provide some variant diversity and hopefully reflect different founder mutations/common mutations in different regions. Lastly, PAM-sites and guide design tend to be variable depending on the target site. As mentioned above, non-canonical PAM-sites appear to work well despite not following strict guidelines. The PAM parameters in this paper were chosen based on recent publications, while aiming to demonstrate the potential flexibility by comparing this to PAMs in the entire guide region.

Whilst there appears to be great opportunity to use base editing to correct one of the disease alleles in a majority of STGD1 patients for therapeutic rescue, a limitation faced by CRISPR systems is the possibility of off-target editing in which undesired nucleotides are edited, yielding potentially detrimental effects. In the early stages of Cas research, these were relatively common, but rapid evolution of Cas based editors has allowed for a notable decline in off-target editing, while maintaining efficient on-target editing (Porto et al., 2020). A prevalent current concern is the effects of bystander editing, where surrounding bases within the guide region are edited alongside the pathogenic mutation. This is of particular concern in *ABCA4*, given the vast number of pathogenic variants. One solution for this is a narrower editing window, which drastically decreases the number of targetable mutations. While this technically reduces the number of targetable mutations, the developing PAM flexibility, and rapid evolution of Cas, will further these possibilities.

One of the main technical limitations faced by nearly all gene editing systems is the delivery mechanism, where only 1% of discovered CRISPR-Cas systems make it into human cells due to size limitations (LaHucik, 2021). Similar to delivery of the *ABCA4* gene, most ABEs and CBEs cannot fit into safer AAV capsid, due to the combined size of the Cas, the deaminase, and gRNA. Many methods have been attempted with varying success to enable efficient delivery to relevant cells. However, a dual vector strategy for separate delivery of the gRNA and the base editor may enable efficient delivery while enabling quick altering of the desired guide. Other delivery methods are discussed in great detail in Cremers et al. (2020) and Piotter et al. (2021). Further it is likely that both delivery methods and construct size will evolve and be improved in the coming years to enable more efficient, safe delivery. For example, recent CRISPR-Cas advances are seen constantly with the discovery of new, small Cas species (Harrington et al., 2018; Karvelis et al., 2020; Schmidt et al., 2021; Xu et al., 2021b). Of particular interest is Cas-MINI, as it has already been tested as an ABE in mammalian cells (but not *in vivo*), shows varying levels of editing efficiency depending on the target site, and easily fits in AAV at only ∼3 kb (Xu et al., 2021b). Alongside the discovery of new CRISPR species, troves of CRISPR ancestors, called IscB proteins, have recently been discovered and identified in a wide diversity of microbes and eukaryotic cells. These systems are much smaller, but have lower editing efficiencies and specific PAM requirements (Altae-Tran et al., 2021). This makes them ideal for few, but specific mutations, with lower risk of off-target, and bystander editing. Similar to prime editing and GBEs, these new Cas systems are exciting, but still require much optimization and elucidation, particularly *in vivo*. Nonetheless, the constant stream of new editing technologies signal a bright future for DNA editing.

Alternative methods to base editing are also being developed, of which prime editing, glycosylase base editors, RNA base editing, and endogenous adenosine deaminase acting on RNA (ADARs) are of particular interest. As previously mentioned, prime editing and GBEs allow for the correction of a broader range of mutation type, but have the need for greater optimization to become more effective (Scholefield and Harrison, 2021). Although DNA editing technologies are enticing due to the potentially curative effect, RNA editing has many advantages, particularly in the lack of PAM-site requirements and the reversibility of the editing, which acts as an added safety. RNA base editors and endogenous ADARs enable PAM-less targeting of RNA to correct G > A transition mutations by A > G editing (Cox et al., 2017; Merkle et al., 2019). However, due to the fact that they target RNA rather than DNA, editing efficiencies may need to be higher in order to see a therapeutic effect, although this is yet to be determined. Further, RNA base editors have high rates of bystander and off-target editing (Cox et al., 2017). Lastly, it is unclear how well RNA base editors work in the retina, as this has not been reported yet. Nonetheless, PAMless RNA editors would enable correction of 32.3% of the total gnomAD variants (1,280/3,979) and 32.8% (227/690) of all *ABCA4* ClinVar variants if further optimized. As with other CRISPR technologies, RNA editing is continually evolving. For example, a newly reported (in BioRxiv) guide-less Pumilio and FBF homology protein RNA base editor, called RNA editing with individual RNA-binding enzyme (REWIRE), achieved a reported 60–80% editing in human cells with little non-specific binding and low levels of off-target effects. Further, in mice intravenously injected with AAV9 and optimized REWIRE systems, 27–34% and 44–51% editing were achieved (Han et al., 2021). With the currently available CRISPR tools or similarly editing molecular tools, and their rate of development, DNA base editors appear to offer the most reproducible, viable editing strategy for correction of *ABCA4* mutations to date.

Finding a functional therapy for Stargardt disease has been a long journey and this study has investigated the potential reach of future CRISPR-base editing treatments. It is highly encouraging that the majority of *ABCA4* mutations are transition mutations and that a large proportion of these have nearby PAM sites, enabling the opportunity for correction. We highlight a roadmap for editing the complex, mutation-rich *ABCA4*, showing the immense potential of using base editors in correcting pathogenic mutations.

## Data Availability

The original contributions presented in the study are included in the article/Supplementary Material, further inquiries can be directed to the corresponding author.
